# NT-proBNP–echocardiographic discordance in type 2 diabetes: links with adiposity and left atrioventricular coupling

**DOI:** 10.3389/fendo.2026.1971124

**Published:** 2026-09-25

**Authors:** Yueyao Zhao, Lina Zhao, Zhourui Liu, Yao Zhang, Jiajue Tan, Nan Chen, Sha Li, Bei Zhang

**Affiliations:** 1 School of Medical Imaging, Guizhou Medical University, Guiyang, Guizhou, China; 2 Ultrasound Center, The Affiliated Hospital of Guizhou Medical University, Guiyang, Guizhou, China

**Keywords:** echocardiography, excess adiposity, left atrial strain, left atrioventricular coupling, NT-ProBNP, triglyceride-glucose index (TyG), type 2 diabetes mellitus

## Abstract

**Background:**

NT-proBNP is widely used to support the exclusion of heart failure, but its concentration may be lower in individuals with excess adiposity and insulin resistance. Whether NT-proBNP below 125 pg/mL excludes early echocardiographic abnormalities in patients with type 2 diabetes mellitus (T2DM) remains uncertain.

**Methods:**

This retrospective cross-sectional study included 191 adults with T2DM hospitalized between May 2024 and November 2025. NT-proBNP measurement and echocardiography were performed within 48 hours. NT-proBNP was categorized at 125 pg/mL and compared with two prespecified echocardiographic outcomes: Layer 1, an operational definition of diastolic dysfunction informed by the 2025 American Society of Echocardiography recommendations and based on consistently available variables; and Layer 2, a composite structural and/or diastolic abnormality. Associations of body mass index (BMI) and the triglyceride-glucose index (TyG) with left atrial and atrioventricular coupling phenotypes were assessed among participants with NT-proBNP <125 pg/mL.

**Results:**

Among the 191 participants, 153 had NT-proBNP <125 pg/mL. Layer 1 abnormality was present in 17 of these participants (11.1%), whereas Layer 2 abnormality was present in 38 (24.8%). Agreement between NT-proBNP classification and Layer 1 and Layer 2 outcomes was 79.6% (Cohen κ=0.326) and 73.3% (Cohen κ=0.328), respectively. In the NT-proBNP <125 pg/mL subgroup, participants with BMI ≥24 kg/m² had higher average E/e′, left atrial volume index, left atrioventricular coupling index, and left atrial stiffness index, and lower left atrial reservoir strain, than those with BMI <24 kg/m². After multivariable adjustment, overweight/obesity was associated with Layer 1 abnormality (odds ratio 3.16, 95% confidence interval 1.03–9.74; P = 0.045) and Layer 2 abnormality (odds ratio 4.15, 95% confidence interval 1.66–10.39; P = 0.002). In mutually adjusted linear models, BMI and TyG were independently associated with higher left atrioventricular coupling index, while TyG was associated with lower left atrial reservoir strain and higher left atrial stiffness index.

**Conclusions:**

In hospitalized adults with T2DM, NT-proBNP below 125 pg/mL did not fully exclude prespecified structural or diastolic echocardiographic abnormalities. Higher BMI was associated with adverse left atrioventricular coupling, whereas a higher TyG index showed more consistent associations with left atrial functional phenotypes. These findings support complementary echocardiographic phenotyping when NT-proBNP concentrations appear reassuring.

## 1 Introduction

Type 2 diabetes mellitus (T2DM) is an established risk factor for heart failure, particularly heart failure with preserved ejection fraction (HFpEF). The transition from metabolic disease to overt heart failure is often preceded by a period of subclinical myocardial involvement: insulin resistance, lipotoxicity, microvascular dysfunction, oxidative stress, low-grade inflammation, and hypertensive loading may progressively impair myocardial relaxation and compliance while left ventricular ejection fraction remains preserved (1–3). During this early phase, tissue Doppler measures, myocardial strain, and left atrial function may become abnormal before typical symptoms or a reduction in ejection fraction. Detecting these changes is therefore important for identifying patients with T2DM who may already have early cardiac involvement despite apparently preserved systolic function.

N-terminal pro-B-type natriuretic peptide (NT-proBNP) is a precursor fragment of brain natriuretic peptide released by cardiomyocytes in response to myocardial wall stress. It is widely used for the initial evaluation, rule-out assessment, and risk stratification of heart failure (4, 5). Its clinical usefulness, however, depends on the extent to which circulating concentration reflects the underlying cardiac phenotype. NT-proBNP is influenced by age, renal function, atrial fibrillation, rhythm, and adiposity, and obesity and insulin-resistant states may suppress circulating natriuretic peptide concentrations. Studies using invasive hemodynamic assessment have shown that some patients with obesity-related HFpEF may have NT-proBNP levels below 125 pg/mL despite elevated resting or exercise-induced left ventricular filling pressures (5–9). Thus, a low value remains clinically informative but may not fully exclude early structural or diastolic abnormalities in patients with T2DM and excess adiposity.

This potential limitation of a single circulating biomarker creates a rationale for complementary echocardiographic phenotyping. Conventional measures, including early diastolic mitral annular velocity (e’), E/e’, left atrial volume index (LAVI), and left ventricular mass index, reflect diastolic function and cumulative loading. Left atrial reservoir strain (LASr) and the left atrial stiffness index (LASI) provide additional information about atrial compliance and pressure burden (11–13). The left atrioventricular coupling index (LACI) integrates atrial and ventricular remodeling through relative volume matching and has been associated with adverse cardiovascular outcomes, including atrial fibrillation and HFpEF (14, 15). Together, these measures can capture different dimensions of early cardiac involvement, including functional impairment, chamber remodeling, and abnormal atrioventricular coupling, even when LVEF and NT-proBNP appear reassuring.

This multimodal perspective is consistent with recent American Heart Association (AHA) statements emphasizing the identification of pre-heart failure before the development of overt symptoms, and with the cardiovascular-kidney-metabolic (CKM) framework, which conceptualizes adiposity, diabetes, insulin resistance, kidney dysfunction, and subclinical cardiovascular disease as interconnected stages of risk (24, 25). Nevertheless, the agreement between NT-proBNP and multidimensional echocardiographic abnormalities in stable hospitalized patients with T2DM remains uncertain, as does the relationship of overweight/obesity and metabolic burden to abnormal atrioventricular coupling among patients with NT-proBNP <125 pg/mL. Accordingly, this study tested two hypotheses: (1) the diagnostic performance of the 125 pg/mL NT-proBNP cutoff for detecting subclinical cardiac abnormalities in hospitalized patients with T2DM is limited, resulting in a subgroup with discordant biomarker and echocardiographic phenotypes; and (2) overweight/obesity and higher insulin resistance are associated with increased LACI and reduced LASr.

## 2 Methods

### 2.1 Study design and participants

This was a single-center retrospective cross-sectional observational study. A total of 191 consecutive patients with T2DM who were hospitalized in the Department of Endocrinology of the Affiliated Hospital of Guizhou Medical University between May 2024 and November 2025 were included. Clinical baseline characteristics, laboratory results, and echocardiographic parameters were collected from the electronic medical record system. All patients underwent conventional echocardiography, two-dimensional speckle-tracking imaging, and three-dimensional echocardiography. The study protocol was approved by the Ethics Committee of the Affiliated Hospital of Guizhou Medical University (approval No. 403). Because this was a retrospective study, the requirement for informed consent was waived by the ethics committee.

### 2.2 Inclusion and exclusion criteria

Inclusion criteria:

- A documented diagnosis of T2DM consistent with the American Diabetes Association diagnostic criteria, based on fasting plasma glucose ≥7.0 mmol/L, 2-h plasma glucose ≥11.1 mmol/L during a 75-g oral glucose tolerance test, glycated hemoglobin ≥6.5%, or, in a patient with classic symptoms of hyperglycemia or hyperglycemic crisis, random plasma glucose ≥11.1 mmol/L (26).
- Age ≥18 years.

Exclusion criteria:

- Congenital heart disease, cardiomyopathy, severe valvular stenosis or regurgitation, atrial fibrillation, other clinically significant arrhythmias, or other relevant cardiac diseases.
- Left ventricular ejection fraction (LVEF) <50% measured using the biplane Simpson method (29).
- Markedly uncontrolled blood pressure, defined as systolic blood pressure >180 mmHg and/or diastolic blood pressure >100 mmHg.
- Poor acoustic window or inadequate image quality that precluded echocardiographic analysis.

### 2.3 Clinical and laboratory data

Demographic and clinical data included sex, age, height, weight, waist circumference, body mass index (BMI), body adiposity index, smoking status, alcohol use, family history of diabetes, diabetes duration, physician-documented hypertension, diabetic retinopathy, diabetic foot disease, and diabetic kidney disease. Laboratory variables included glycated hemoglobin (HbA1c), fasting plasma glucose (FPG), triglycerides (TG), total cholesterol, high-density lipoprotein cholesterol (HDL-C), low-density lipoprotein cholesterol, N-terminal pro-B-type natriuretic peptide (NT-proBNP), uric acid, serum creatinine, alanine aminotransferase, and aspartate aminotransferase. The first available laboratory measurements after admission were used. Medication exposure at the index admission was recorded for sodium-glucose cotransporter 2 inhibitors, glucagon-like peptide-1 receptor agonists, insulin, metformin, dipeptidyl peptidase-4 inhibitors, alpha-glucosidase inhibitors, other glucose-lowering agents, renin-angiotensin system inhibitors, beta-blockers, calcium channel blockers, diuretics or mineralocorticoid receptor antagonists, statins, and antiplatelet agents.

BMI was calculated from measured weight and height. BMI ≥24 kg/m² was prespecified as overweight/obesity, and BMI <24 kg/m² was the reference category, consistent with the recommended cutoff for Chinese adults (27). The triglyceride-glucose (TyG) index and metabolic score for insulin resistance (METS-IR) were calculated using the published equations (16, 17). When glucose and lipid concentrations were reported in mmol/L, FPG was multiplied by 18, TG by 88.6, and HDL-C by 38.67 before calculation.


$$BMI\;=\frac{weight\;(kg)}{height^{2}(m^{2})}$$

$$TyG\;=\;ln[\frac{TG\;(mg/dL)\;\times \;FPG\;(mg/dL)}{2}]$$

$$METS-IR=\frac{ln[2\times FPG\;(mg/dL)+TG\;(mg/dL)]\times BMI}{ln[HDL-C\;(mg/dL)]}$$

Estimated glomerular filtration rate (eGFR) was calculated using the 2021 race-free Chronic Kidney Disease Epidemiology Collaboration creatinine equation (28). Serum creatinine was converted from μmol/L to mg/dL by division by 88.4. In the equation below, κ was 0.7 for women and 0.9 for men, and α was −0.241 for women and −0.302 for men.


$$eGFR=142\times min{\left(Scr/\kappa ,1\right)}^{\alpha }\times max{\left(Scr/\kappa ,\;1\right)}^{-1.200}\times 0.9938^{Age}\times 1.012\;(if\;female)$$

The resulting eGFR was expressed in mL/min/1.73 m².

### 2.4 Echocardiography

Echocardiographic examinations were performed using Philips CVX and EPIQ 7C systems equipped with an X5–1 matrix-array transducer. Participants were examined in the left lateral decubitus position with simultaneous electrocardiographic monitoring. Dynamic images from five consecutive cardiac cycles were acquired at end-expiration and stored for offline analysis. Conventional two-dimensional and Doppler parameters included left atrial diameter, left ventricular end-diastolic diameter, interventricular septal thickness, peak early (E) and late (A) diastolic transmitral inflow velocities, the E/A ratio, lateral and septal mitral annular e′ velocities, average e′, and average E/e′. LVEF was measured using the biplane Simpson method. Left ventricular mass (LVM) was derived from two-dimensional measurements, and left ventricular mass index (LVMI) was calculated by indexing LVM to body surface area in accordance with chamber quantification recommendations (29).

Two-dimensional speckle-tracking analysis was performed offline using the TomTec AutoStrain LV/LA/RV modules to obtain left ventricular global longitudinal strain, left atrial reservoir strain (LASr), conduit strain, contraction strain, and right ventricular free-wall longitudinal strain according to current deformation-imaging recommendations (11). The left atrial stiffness index (LASI) was calculated as average E/e′ divided by LASr. Three-dimensional datasets were analyzed using the corresponding TomTec modules to obtain three-dimensional global longitudinal strain, three-dimensional global circumferential strain, left ventricular end-diastolic volume (LVEDV), left ventricular end-systolic volume, stroke volume, cardiac index, maximum and minimum left atrial volumes, left atrial emptying fraction, left atrial volume index (LAVI), right ventricular end-diastolic volume, and right ventricular end-systolic volume. Cardiac volumes were indexed to body surface area where appropriate. The left atrioventricular coupling index (LACI) was calculated as three-dimensional minimum left atrial volume divided by three-dimensional LVEDV (14, 15).

Repeatability was assessed in 30 randomly selected participants using intraclass correlation coefficients (ICCs) from a two-way absolute-agreement model and Bland-Altman analysis. Intraobserver measurements were repeated after at least 2 weeks, and interobserver measurements were obtained independently from the same stored images.

### 2.5 Definition of echocardiographic outcomes

The two-layer outcome framework and the thresholds used in the current analysis were defined before analysis of the final dataset and were applied consistently to all participants. The Layer 1 outcome represented guideline-informed diastolic dysfunction. Its definition was adapted from the 2025 American Society of Echocardiography recommendations using echocardiographic variables consistently available in this retrospective dataset (10). Reduced mitral annular e′ velocity was defined as lateral e′ ≤7 cm/s, septal e′ ≤6 cm/s, or average e′ ≤6.5 cm/s. Layer 1 abnormality was defined as reduced e′ accompanied by at least one additional abnormal parameter (average E/e′ >14, two-dimensional left atrial reservoir strain [LASr] ≤18%, or left atrial volume index [LAVI] >34 mL/m²), or preserved e′ accompanied by at least two of these abnormal parameters. Tricuspid regurgitation velocity and pulmonary artery systolic pressure were not consistently available and therefore could not be incorporated. The mitral E/A ratio was recorded as a descriptive parameter but was not included in the Layer 1 definition because it is influenced by age and may exhibit pseudonormalization.

The Layer 2 outcome was a composite structural and/or diastolic abnormality, defined as the presence of Layer 1 abnormality, LAVI >34 mL/m², or elevated two-dimensional left ventricular mass index (LVMI; >95 g/m² in women and >115 g/m² in men) (29). Because participants with left ventricular ejection fraction <50% were excluded, the Layer 2 outcome primarily represented structural and/or diastolic abnormalities in patients with preserved systolic function. Left ventricular global longitudinal strain, the left atrioventricular coupling index, and the left atrial stiffness index were not included in the composite outcome because they are subclinical or derived indices without uniformly accepted diagnostic thresholds. These measures were analyzed separately as continuous echocardiographic phenotypes.

### 2.6 Statistical analysis

Continuous variables are presented as median (lower quartile–upper quartile), and categorical variables as number (percentage). Comparisons between groups were performed using the Mann–Whitney U test for continuous variables and the Pearson χ² test or Fisher exact test for categorical variables, as appropriate. Rank-biserial correlations and standardized differences in proportions were reported as effect sizes. Agreement between NT-proBNP classification and the two echocardiographic outcomes was assessed using overall agreement and Cohen κ. Sensitivity, specificity, positive predictive value, and negative predictive value were reported with Wilson 95% confidence intervals.

Spearman rank correlation analysis was used to examine associations between metabolic indices and echocardiographic parameters. Firth penalized logistic regression was used for binary echocardiographic outcomes (30), and multivariable linear regression with HC3 heteroskedasticity-robust standard errors was used for continuous echocardiographic outcomes. Prespecified sensitivity analyses evaluated the potential influence of medication use, NT-proBNP level, and impaired renal function. Intraobserver and interobserver reproducibility was evaluated using intraclass correlation coefficients with 95% confidence intervals and Bland–Altman analysis. The Benjamini–Hochberg procedure was applied to control the false discovery rate where multiple comparisons were performed (31). All tests were two-sided, and P<0.05 was considered statistically significant. Statistical analyses were conducted using Python version 3.12.10 with the pandas, SciPy, and statsmodels packages.

## 3 Results

### 3.1 NT-proBNP classification and dual-layer echocardiographic outcomes

Among 191 participants, 153 had NT-proBNP <125 pg/mL and 38 had NT-proBNP ≥125 pg/mL. Layer 1 abnormality was present in 33 participants, including 17 of 153 (11.1%) with NT-proBNP <125 pg/mL and 16 of 38 (42.1%) with NT-proBNP ≥125 pg/mL. Layer 2 abnormality was present in 63 participants, including 38 of 153 (24.8%) and 25 of 38 (65.8%) in the respective NT-proBNP groups (**Figure 1**).

**Figure 1:**
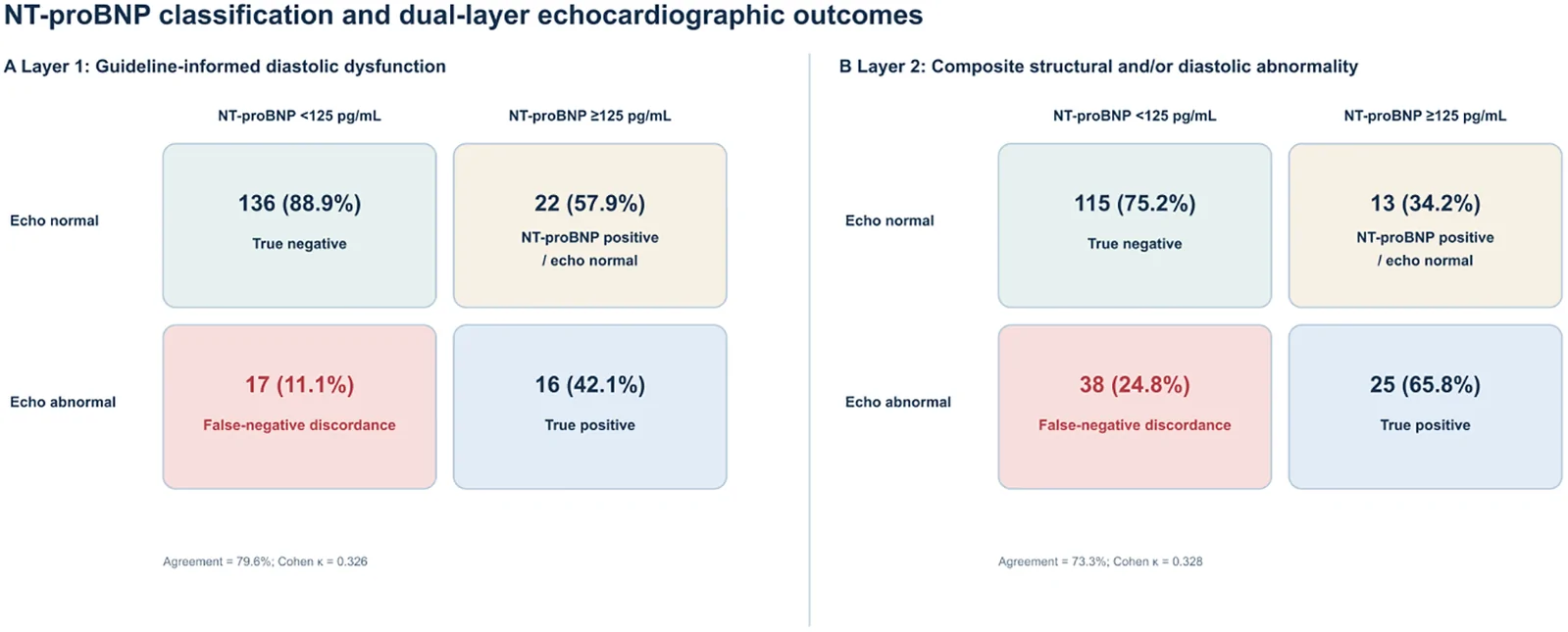
NT-proBNP classification and dual-layer echocardiographic outcomes. **(A)** Layer 1: guideline-informed diastolic dysfunction. **(B)** Layer 2: composite structural and/or diastolic abnormality. Each cell reports n (% within the corresponding NT-proBNP group); percentages in each column sum to 100%. Layer definitions were prespecified in the Methods.

At the 125-pg/mL threshold, NT-proBNP had a sensitivity of 48.5%, specificity of 86.1%, positive predictive value of 42.1%, and negative predictive value of 88.9% for Layer 1 abnormality. The corresponding estimates for Layer 2 abnormality were 39.7%, 89.8%, 65.8%, and 75.2%, respectively. Overall agreement was 79.6% for Layer 1 and 73.3% for Layer 2 (**Table 1**).

**Table 1:** Diagnostic performance of NT-proBNP >=125 pg/mL for the dual-layer echocardiographic outcomes.

Outcome	Sensitivity	Specificity	Positive predictive value	Negative predictive value	Overall agreement	Cohen kappa
Layer 1: guideline-informed diastolic dysfunction	48.5% (32.5%–64.8%)	86.1% (79.8%–90.6%)	42.1% (27.9%–57.8%)	88.9% (82.9%–92.9%)	79.6%	0.326
Layer 2: Composite structural and/or diastolic abnormality	39.7% (28.5%–52.0%)	89.8% (83.4%–94.0%)	65.8% (49.9%–78.8%)	75.2% (67.8%–81.3%)	73.3%	0.328

Values in parentheses are Wilson 95% confidence intervals. NT-proBNP, N-terminal pro-B-type natriuretic peptide.

Component-level analysis showed that left atrial volume index elevation was the most frequent component of the Layer 2 outcome, occurring in 46 of 63 patients (73.0%), followed by Layer 1 abnormality in 33 (52.4%) and elevated left ventricular mass index in 22 (34.9%). Overall, 30 of 63 patients (47.6%) did not meet the Layer 1 definition and were identified through left atrial volume index and/or left ventricular mass index abnormalities. Among the 38 patients with NT-proBNP <125 pg/mL and Layer 2 abnormality, 21 (55.3%) had structural abnormalities without concomitant Layer 1 dysfunction ( **Supplementary Table S2**).

### 3.2 Reproducibility of echocardiographic measurements

Intraobserver ICCs ranged from 0.855 to 0.989, and interobserver ICCs ranged from 0.855 to 0.983. Agreement was highest for three-dimensional left atrial volume measurements and LACI; average E/e′ and LASI had the lowest interobserver ICCs (0.873 and 0.855, respectively). Bland-Altman analyses showed small mean biases without a consistent directional pattern across measurements ( **Supplementary Table S1**, **Supplementary Figure S1**).

### 3.3 Clinical, metabolic, and echocardiographic phenotypes according to BMI

Among patients with NT-proBNP <125 pg/mL, 67 had a BMI <24 kg/m² and 86 had a BMI ≥24 kg/m². Compared with the lower-BMI group, the higher-BMI group had a shorter diabetes duration [4.5 (1.0–10.0) vs 9.0 (3.0–18.0) years; P = 0.039], a larger waist circumference [92.5 (86.0–97.0) vs 83.0 (78.0–88.0) cm; P<0.001], a higher triglyceride-glucose index [9.57 (9.00–10.07) vs 9.14 (8.79–9.79); P = 0.040], and a higher metabolic score for insulin resistance [44.84 (40.84–50.08) vs 35.71 (33.16–38.38); P<0.001].

Echocardiographically, the higher-BMI group had a higher average E/e′ ratio [9.10 (8.00–10.37) vs 8.56 (6.98–9.62); P = 0.008], a larger left atrial volume index [28.4 (22.8–33.9) vs 26.3 (21.8–30.0) mL/m²; P = 0.029], a lower left atrial reservoir strain [33.0 (26.0–39.1)% vs 37.1 (30.1–48.0)%; P = 0.015], a higher left atrioventricular coupling index [0.21 (0.17–0.26) vs 0.17 (0.14–0.19); P<0.001], and a higher left atrial stiffness index [0.28 (0.23–0.36) vs 0.23 (0.17–0.31); P<0.001]. Estimated glomerular filtration rate, glycated hemoglobin, NT-proBNP, Simpson left ventricular ejection fraction, longitudinal and circumferential strain, and left ventricular mass index did not differ significantly between the groups (**Table 2**).

**Table 2:** Clinical, metabolic, echocardiographic, and selected treatment characteristics of patients with NT-proBNP <125 pg/mL stratified by BMI.

Variable	Body mass index <24 kg/m2 (n=67)	Body mass index >=24 kg/m2 (n=86)	P value	Effect size
Clinical and laboratory characteristics				
Age, years	59.0 (50.0–66.5)	56.0 (43.8–64.8)	0.227	-0.11
Male sex	45 (67.2)	61 (70.9)	0.616	0.08
Diabetes duration, years	9.0 (3.0–18.0)	4.5 (1.0–10.0)	0.039	-0.19
SBP, mmHg	121.0 (113.0–134.0)	124.5 (115.2–134.0)	0.419	0.08
DBP, mmHg	79.0 (71.0–86.5)	79.0 (74.0–88.8)	0.503	0.06
WC, cm	83.0 (78.0–88.0)	92.5 (86.0–97.0)	<0.001	0.60
Hypertension	20 (29.9)	38 (44.2)	0.070	0.30
HbA1c, %	9.30 (7.05–11.05)	8.31 (6.89–10.59)	0.264	-0.11
TyG	9.14 (8.79–9.79)	9.57 (9.00–10.07)	0.040	0.19
METS-IR	35.71 (33.16–38.38)	44.84 (40.84–50.08)	<0.001	0.78
eGFR, mL/min/1.73 m²	100.1 (87.1–108.8)	101.8 (88.6–111.4)	0.350	0.09
NT-proBNP, pg/mL	52.0 (35.0–66.0)	43.5 (33.0–62.8)	0.330	-0.09
Echocardiographic characteristics				
LVEF by Simpson method, %	63.0 (59.0–65.0)	62.5 (59.0–65.0)	0.693	-0.04
Average E/e′	8.56 (6.98–9.62)	9.10 (8.00–10.37)	0.008	0.25
2D GLS, %	-18.3 (-20.7–15.9)	-17.8 (-20.3–15.6)	0.521	0.06
3D GLS, %	-24.6 (-27.9–20.8)	-25.8 (-28.9–21.7)	0.282	-0.10
3D GCS, %	-27.7 (-30.4–25.7)	-26.7 (-30.7–25.0)	0.411	0.08
2D LVMI, g/m²	78.4 (68.1–89.3)	81.6 (70.1–93.3)	0.408	0.08
3D LAVI, mL/m²	26.3 (21.8–30.0)	28.4 (22.8–33.9)	0.029	0.21
2D LASr, %	37.1 (30.1–48.0)	33.0 (26.0–39.1)	0.015	-0.23
LACI	0.17 (0.14–0.19)	0.21 (0.17–0.26)	<0.001	0.43
LASI	0.23 (0.17–0.31)	0.28 (0.23–0.36)	<0.001	0.36
Selected therapies				
SGLT2 inhibitor	30 (44.8)	45 (52.3)	0.354	0.15
GLP-1 receptor agonist	6 (9.0)	35 (40.7)	<0.001	0.79
Insulin	42 (62.7)	37 (43.0)	0.016	-0.40
Metformin	40 (59.7)	53 (61.6)	0.809	0.04
RAS inhibitor	13 (19.4)	21 (24.4)	0.459	0.12
Diuretic or MRA	2 (3.0)	0 (0.0)	0.190	-0.25

Continuous variables are median (lower quartile-upper quartile); categorical variables are n (%). P values are unadjusted. BMI, body mass index; SBP, systolic blood pressure; DBP, diastolic blood pressure; WC, waist circumference; HbA1c, glycated hemoglobin; TyG, triglyceride-glucose index; METS-IR, metabolic score for insulin resistance; eGFR, estimated glomerular filtration rate; NT-proBNP, N-terminal pro-B-type natriuretic peptide; LVEF, left ventricular ejection fraction; E/e′, average transmitral E/e′ ratio; 2D, two-dimensional; 3D, three-dimensional; GLS, global longitudinal strain; GCS, global circumferential strain; LVMI, left ventricular mass index; LAVI, left atrial volume index; LASr, left atrial reservoir strain; LACI, left atrioventricular coupling index; LASI, left atrial stiffness index; SGLT2, sodium-glucose cotransporter 2; GLP-1, glucagon-like peptide-1; RAS, renin-angiotensin system; MRA, mineralocorticoid receptor antagonist.

Distributional analysis was consistent with the group summaries. Compared with patients with BMI <24 kg/m², those with BMI ≥24 kg/m² had a higher left atrioventricular coupling index (rank-biserial correlation=0.43; P<0.001), lower two-dimensional left atrial reservoir strain (rank-biserial correlation=−0.23; P = 0.015), and a higher left atrial stiffness index (rank-biserial correlation=0.36; P<0.001) (**Figure 2**).

**Figure 2:**
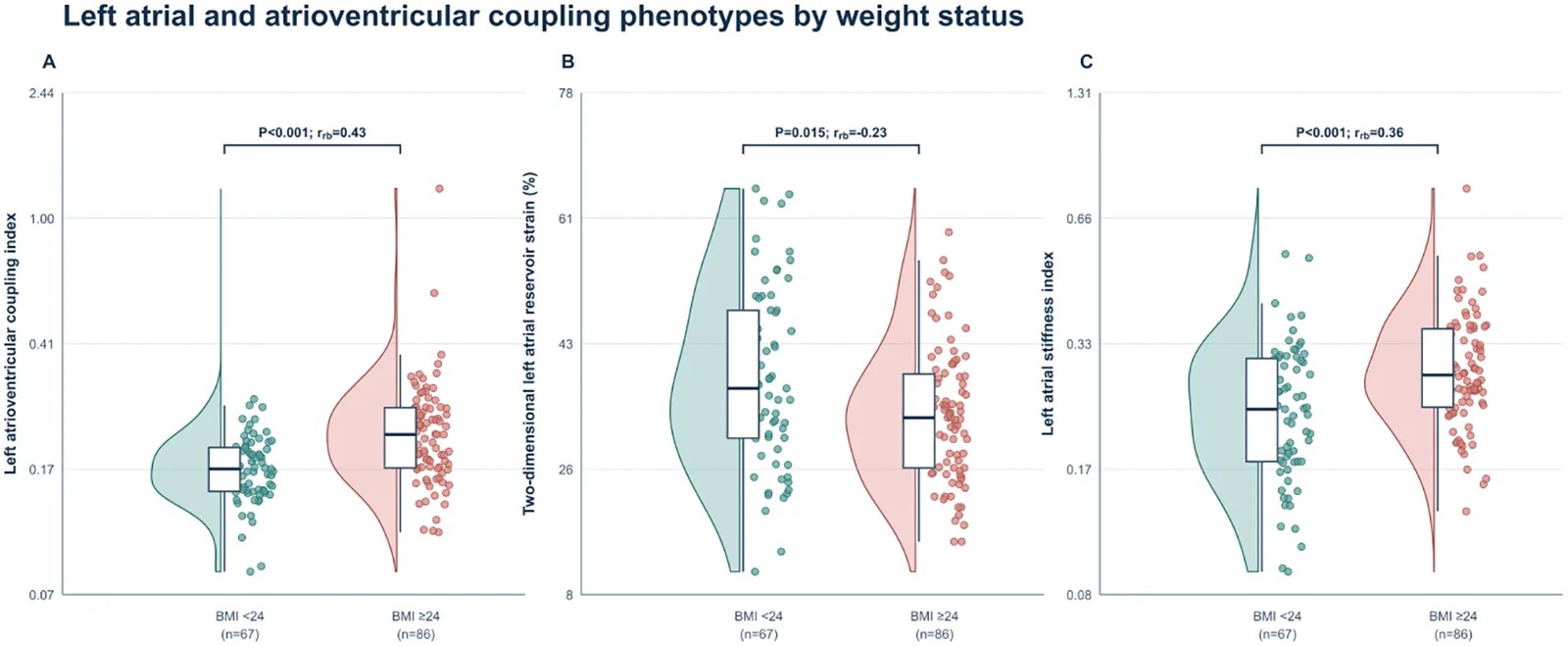
Left atrial and atrioventricular coupling phenotypes by weight status in patients with NT-proBNP <125 pg/mL. **(A)** Left atrioventricular coupling index (LACI); **(B)** two-dimensional left atrial reservoir strain (LASr); **(C)** left atrial stiffness index (LASI). BMI <24 kg/m^2^, n=67; BMI ≥24 kg/m^2^, n=86. Half-violin plots show distributions, box plots show medians and interquartile ranges, and points indicate individual observations. LACI and LASI are shown on logarithmic scales. P values are from Mann–Whitney U tests; r_b denotes the rank-biserial correlation.

### 3.4 Association of overweight/obesity with dual-layer echocardiographic outcomes

In Firth logistic regression restricted to patients with NT-proBNP <125 pg/mL, overweight/obesity was associated with Layer 1 abnormality after adjustment for age, sex, diabetes duration, hypertension, estimated glomerular filtration rate, and glycated hemoglobin (odds ratio 3.16, 95% confidence interval 1.03–9.74; P = 0.045). For Layer 2 abnormality, the corresponding odds ratio was 4.15 (95% confidence interval 1.66–10.39; P = 0.002) (**Figure 3**).

**Figure 3:**
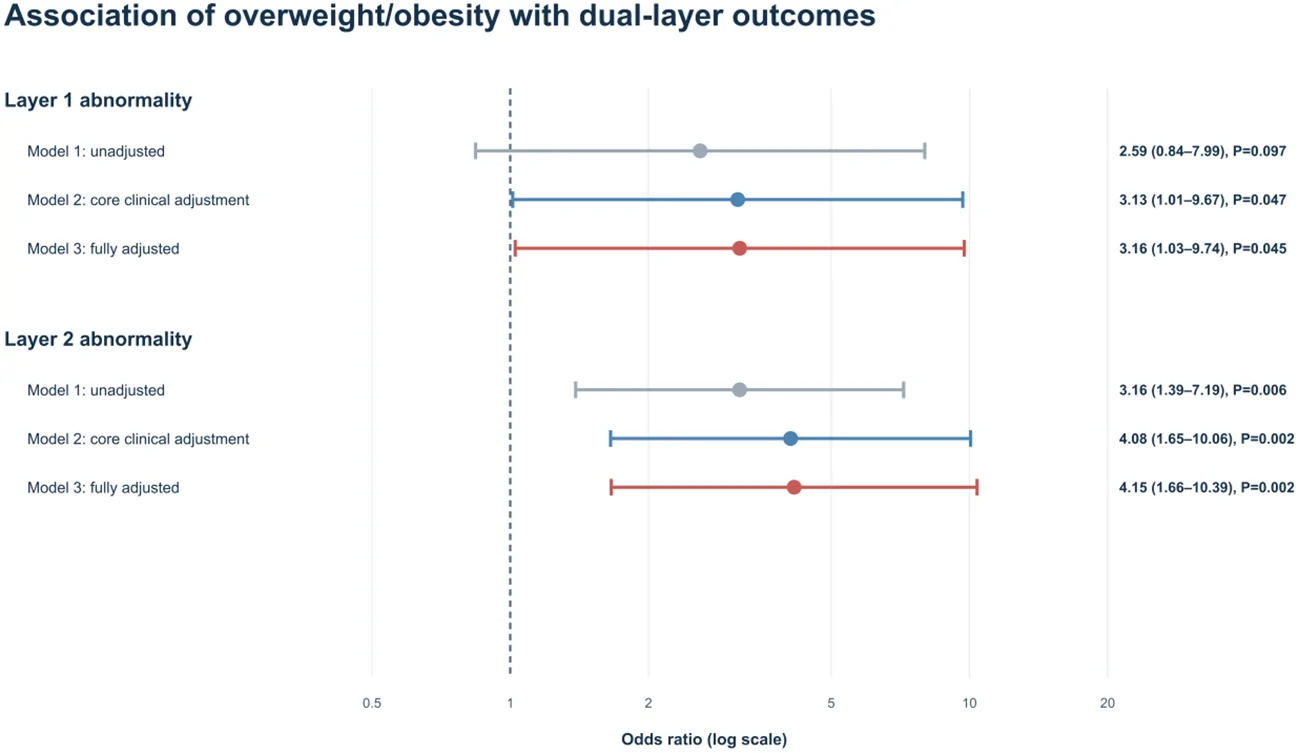
Firth logistic regression of overweight/obesity and dual-layer echocardiographic outcomes in patients with NT-proBNP <125 pg/mL. Model 1 was unadjusted; Model 2 included age, sex, diabetes duration, and hypertension; Model 3 additionally included eGFR and HbA1c. The reference group was BMI <24 kg/m². Points are odds ratios and horizontal lines are 95% confidence intervals.

### 3.5 Metabolic–echocardiographic relationships

Spearman correlation analysis identified the strongest metabolic–echocardiographic association between the metabolic score for insulin resistance and the left atrioventricular coupling index (ρ=0.53; false-discovery-rate-adjusted q<0.001). The metabolic score for insulin resistance was also correlated with the left atrial stiffness index (ρ=0.41; q<0.001) and inversely correlated with two-dimensional left atrial reservoir strain (ρ=−0.30; q=0.001). Body mass index and the triglyceride-glucose index were positively correlated with the left atrioventricular coupling index (ρ=0.39 and 0.34, respectively; both q<0.001) (**Figure 4**).

**Figure 4:**
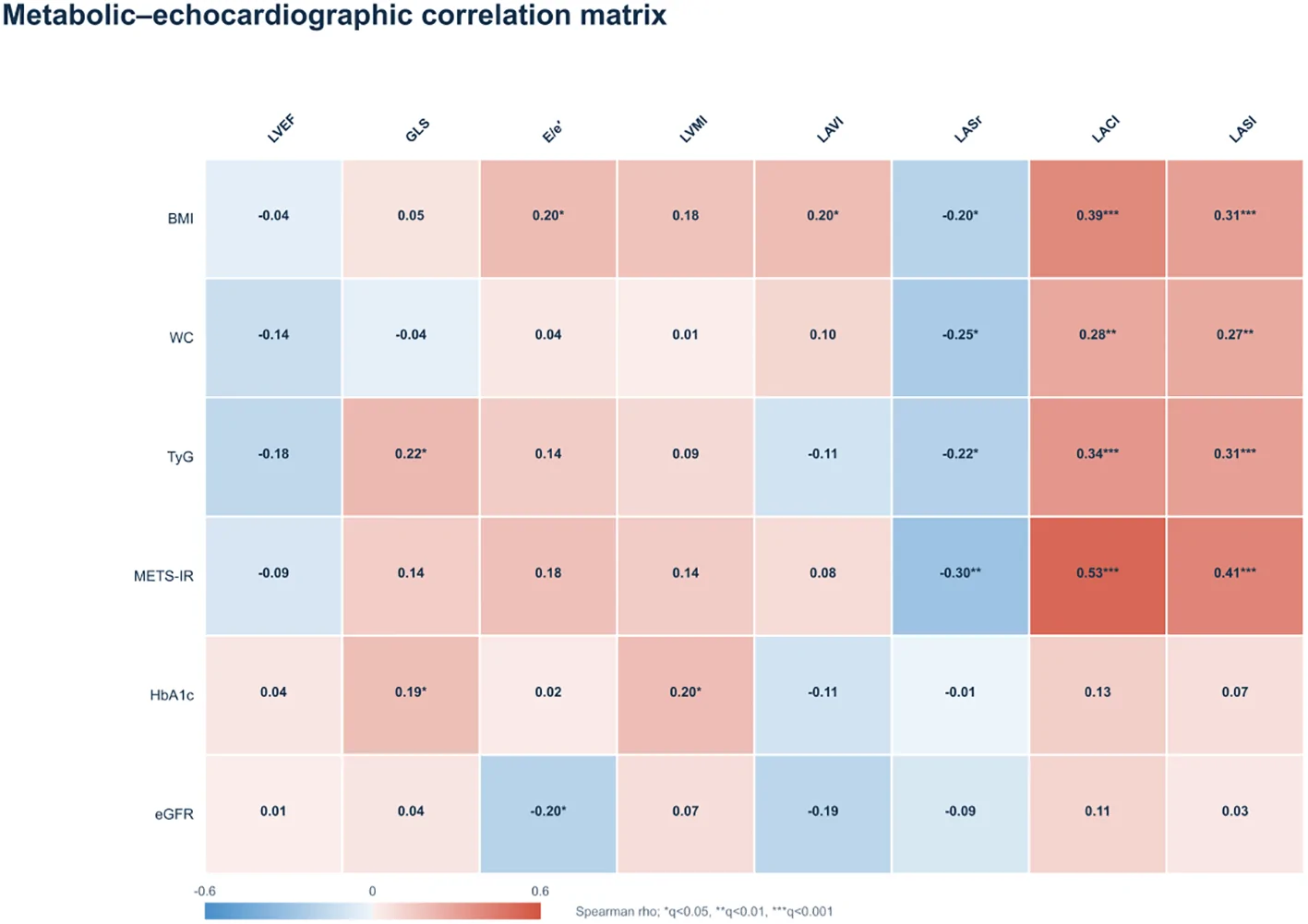
Metabolic-echocardiographic correlation matrix in patients with NT-proBNP <125 pg/mL. Cells show Spearman correlation coefficients; q values were adjusted across the displayed matrix using the Benjamini-Hochberg procedure. *q<0.05, **q<0.01, and ***q<0.001. BMI, body mass index; WC, waist circumference; TyG, triglyceride-glucose index; METS-IR, metabolic score for insulin resistance; HbA1c, glycated hemoglobin; eGFR, estimated glomerular filtration rate; LVEF, Simpson left ventricular ejection fraction; GLS, global longitudinal strain; GCS, global circumferential strain; LACI, left atrioventricular coupling index; LASI, left atrial stiffness index; LASr, left atrial reservoir strain; LAVI, left atrial volume index; LVMI, left ventricular mass index.

Among patients with NT-proBNP <125 pg/mL (n=153), sequential multivariable linear regression models were progressively adjusted for age and sex (Model 1), diabetes duration and hypertension (Model 2), and estimated glomerular filtration rate and glycated hemoglobin (Model 3). The associations of body mass index and the triglyceride-glucose index with the left atrioventricular coupling index remained positive across Models 1-3. In the mutually adjusted Model 4, which included body mass index and the triglyceride-glucose index simultaneously with all covariates, higher body mass index (standardized beta=0.22, 95% confidence interval 0.09-0.34; P<0.001; q=0.001) and a higher triglyceride-glucose index (standardized beta=0.17, 95% confidence interval 0.06-0.28; P = 0.002; q=0.002) were independently associated with a higher left atrioventricular coupling index. For two-dimensional left atrial reservoir strain, the inverse association with the triglyceride-glucose index persisted after full adjustment (standardized beta=-0.26, 95% confidence interval -0.46 to -0.06; P = 0.010; q=0.027), whereas the corresponding association with body mass index was not statistically significant (standardized beta=-0.10, 95% confidence interval -0.27 to 0.07; P = 0.244; q=0.244). For the left atrial stiffness index, the triglyceride-glucose index remained positively associated (standardized beta=0.29, 95% confidence interval 0.10-0.48; P = 0.003; q=0.005), while the body mass index association did not meet the false-discovery-rate-adjusted threshold (standardized beta=0.16, 95% confidence interval -0.00 to 0.32; P = 0.057; q=0.057) (**Table 3**).

**Table 3:** Sequential multivariable linear regression of metabolic indices and left atrial phenotypes among patients with NT-proBNP <125 pg/mL.

Outcome	Model	Exposure	n	Standardized β (95% CI)	P value	Adjusted q value	Model R²
LACI	Model 1: age + sex	BMI	153	0.26 (0.13 to 0.38)	<0.001	<0.001	0.074
		TyG	153	0.22 (0.11 to 0.34)	<0.001	<0.001	0.056
	Model 2: Model 1 + diabetes duration + hypertension	BMI	153	0.25 (0.12 to 0.39)	<0.001	<0.001	0.076
		TyG	153	0.22 (0.11 to 0.33)	<0.001	<0.001	0.062
	Model 3: Model 2 + eGFR + HbA1c	BMI	153	0.25 (0.12 to 0.38)	<0.001	<0.001	0.116
		TyG	153	0.23 (0.11 to 0.34)	<0.001	<0.001	0.096
	Model 4: Model 3 + BMI + TyG (mutually adjusted)	BMI	153	0.22 (0.09 to 0.34)	<0.001	<0.001	0.138
		TyG	153	0.17 (0.06 to 0.28)	0.002	0.002	0.138
LASr	Model 1: age + sex	BMI	153	-0.17 (-0.33 to -0.01)	0.037	0.060	0.092
		TyG	153	-0.21 (-0.37 to -0.05)	0.009	0.027	0.107
	Model 2: Model 1 + diabetes duration + hypertension	BMI	153	-0.16 (-0.32 to 0.01)	0.069	0.087	0.101
		TyG	153	-0.20 (-0.36 to -0.04)	0.015	0.029	0.117
	Model 3: Model 2 + eGFR + HbA1c	BMI	153	-0.15 (-0.32 to 0.02)	0.076	0.087	0.104
		TyG	153	-0.28 (-0.47 to -0.10)	0.002	0.019	0.143
	Model 4: Model 3 + BMI + TyG (mutually adjusted)	BMI	153	-0.10 (-0.27 to 0.07)	0.244	0.244	0.153
		TyG	153	-0.26 (-0.46 to -0.06)	0.010	0.027	0.153
LASI	Model 1: age + sex	BMI	153	0.23 (0.07 to 0.38)	0.005	0.008	0.067
		TyG	153	0.29 (0.13 to 0.45)	<0.001	0.002	0.097
	Model 2: Model 1 + diabetes duration + hypertension	BMI	153	0.21 (0.05 to 0.37)	0.012	0.015	0.102
		TyG	153	0.27 (0.12 to 0.42)	<0.001	0.002	0.131
	Model 3: Model 2 + eGFR + HbA1c	BMI	153	0.21 (0.05 to 0.38)	0.013	0.015	0.104
		TyG	153	0.33 (0.15 to 0.50)	<0.001	0.002	0.143
	Model 4: Model 3 + BMI + TyG (mutually adjusted)	BMI	153	0.16 (-0.00 to 0.32)	0.057	0.057	0.164
		TyG	153	0.29 (0.10 to 0.47)	0.003	0.005	0.164

Model 1 included age and sex; Model 2 additionally included diabetes duration and hypertension; Model 3 additionally included eGFR and HbA1c; Model 4 mutually adjusted BMI and TyG with all Model 3 covariates. Continuous outcomes and exposures were standardized; HC3 robust standard errors were used. q values were adjusted within each outcome using the Benjamini-Hochberg procedure. BMI, body mass index; CI, confidence interval; eGFR, estimated glomerular filtration rate; HbA1c, glycated hemoglobin; LACI, left atrioventricular coupling index; LASI, left atrial stiffness index; LASr, left atrial reservoir strain; NT-proBNP, N-terminal pro-B-type natriuretic peptide; TyG, triglyceride-glucose index.

### 3.6 Treatment patterns and sensitivity analyses

Medication use is summarized in **Supplementary Table S3**. In the overall cohort, patients with NT-proBNP >=125 pg/mL were less frequently treated with glucagon-like peptide-1 receptor agonists (4/38 [10.5%] vs 41/153 [26.8%]; P = 0.034), metformin (15/38 [39.5%] vs 93/153 [60.8%]; P = 0.018), and dipeptidyl peptidase-4 inhibitors (2/38 [5.3%] vs 29/153 [19.0%]; P = 0.041), and more frequently received a diuretic or mineralocorticoid receptor antagonist (6/38 [15.8%] vs 2/153 [1.3%]; P<0.001). Within the NT-proBNP <125 pg/mL subgroup, patients with BMI >=24 kg/m2 more frequently received glucagon-like peptide-1 receptor agonists (35/86 [40.7%] vs 6/67 [9.0%]; P<0.001) and less frequently received insulin (37/86 [43.0%] vs 42/67 [62.7%]; P = 0.016) or an alpha-glucosidase inhibitor (10/86 [11.6%] vs 19/67 [28.4%]; P = 0.009). These comparisons were unadjusted and descriptive; the remaining medication comparisons were not statistically significant. In **Supplementary Table S4**, the association between overweight/obesity and Layer 2 abnormality was stable after separate additional adjustment for log10-transformed NT-proBNP and each prespecified therapy. The adjusted odds ratios ranged from 4.19 to 4.99, with corresponding P values ranging from 0.001 to 0.0024. After exclusion of participants with estimated glomerular filtration rate <60 mL/min/1.73 m2, the association remained positive (odds ratio 4.10, 95% confidence interval 1.54-10.88; P = 0.005). For Layer 1 abnormality, point estimates remained above 3.0 in the medication-adjusted models, although confidence intervals were wider and some P values crossed 0.05; the renal-exclusion analysis yielded an odds ratio of 2.85 (95% confidence interval 0.91-8.96; P = 0.073). In medication-adjusted continuous-outcome models, body mass index and the triglyceride-glucose index remained positively associated with the left atrioventricular coupling index. The triglyceride-glucose index also remained inversely associated with two-dimensional left atrial reservoir strain and positively associated with the left atrial stiffness index; these directions were unchanged after additional NT-proBNP adjustment and after exclusion of reduced renal function (**Supplementary Table S4**).

## 4 Discussion

### 4.1 Prevalence and screening interpretation of discordance between NT-proBNP and cardiac echocardiographic phenotypes

In this study of 191 stable hospitalized patients with T2DM, NT-proBNP classification at 125 pg/mL showed only modest agreement with the two prespecified echocardiographic outcomes. Even among patients with NT-proBNP <125 pg/mL, 17 of 153 (11.1%) met the Layer 1 definition and 38 of 153 (24.8%) had Layer 2 structural and/or diastolic abnormalities. These findings indicate that NT-proBNP <125 pg/mL may exclude many, but not all, echocardiographic abnormalities in this population.

This discordance does not imply that NT-proBNP lacks clinical value. NT-proBNP primarily reflects circulating myocardial wall stress at the time of measurement, whereas LAVI, LVMI, e’, and atrioventricular coupling parameters also capture cumulative loading, remodeling, and impaired compliance. Because invasive left ventricular filling pressure and prospective clinical outcomes were not used as reference standards, the observed pattern is more appropriately described as biomarker-imaging phenotypic discordance rather than conventional false-negative heart failure. The presence of structural or diastolic abnormalities in patients with low NT-proBNP is compatible with a pre-heart failure phenotype, but does not establish a formal diagnosis of AHA Stage B heart failure (24). This interpretation is consistent with studies of obesity-related HFpEF in which filling pressures may be abnormal despite relatively low natriuretic peptide concentrations (6–9, 18).

### 4.2 Potential pathophysiological mechanisms linking obesity and metabolic burden to abnormal left atrioventricular coupling

The weight-related differences observed in this study provide a possible explanation for the biomarker-imaging discordance. Even without overt NT-proBNP elevation, patients with BMI >=24 kg/m2 had higher LACI, LAVI, E/e’, and LASI and lower LASr, together with larger waist circumference and higher TyG and METS-IR. Similar impairments in left atrial strain have been described in patients with diabetes and obesity and in middle-aged adults with obesity (20, 21). Previous studies have shown that obesity and insulin resistance are associated with lower circulating natriuretic peptide concentrations (6, 8, 9, 18, 19). Visceral adiposity may be particularly relevant because regional fat distribution can influence natriuretic peptide levels beyond BMI alone (19). Thus, BMI in this study may represent both adiposity burden and a broader metabolic phenotype, whereas waist circumference provides additional, although indirect, information about central adiposity.

Left atrial and atrioventricular coupling indices may detect early metabolic cardiac involvement before conventional systolic indices become abnormal. LASr reflects left atrial reservoir function and is influenced by left ventricular longitudinal function, atrial compliance, and filling pressure. LASI combines E/e’ and LASr and may reflect pressure load relative to deformation capacity. LACI captures adverse left atrial-left ventricular volume matching. In the present study, LACI and LASI differed significantly between BMI groups, whereas Simpson LVEF, GLS, LVMI, and other conventional systolic measures did not. This pattern supports a phenotype characterized predominantly by atrial functional and coupling abnormalities rather than overt systolic impairment, although the cross-sectional design cannot establish the temporal sequence of these changes.

### 4.3 Metabolic burden and independent associations with left atrial phenotypes

The regression analyses provide complementary evidence that the observed phenotype was not explained solely by age, sex, diabetes duration, hypertension, renal function, or glycemic control. BMI and TyG were each positively associated with LACI across sequential models, and both remained independently associated with LACI in the mutually adjusted model. In contrast, the inverse association between TyG and two-dimensional LASr persisted after mutual adjustment, whereas the BMI association with LASr was attenuated and no longer statistically significant. TyG was also independently associated with higher LASI, while the BMI association became borderline after TyG adjustment. This pattern helps distinguish the information carried by structural and functional indices. BMI showed a modest correlation with LAVI (rho=0.20; q=0.040), but not with LVMI after false-discovery-rate adjustment (rho=0.18; q=0.065), and the present study did not fit independent regression models with LAVI or LVMI as continuous outcomes. The strongest BMI signal was observed for LACI (rho=0.39; q<0.001), an integrative index of atrioventricular volume matching that may be sensitive to early functional coupling changes. By contrast, LAVI and LVMI are cumulative, threshold-based structural measures that may require a longer or more intense exposure to produce measurable remodeling and may be influenced by blood pressure, diabetes duration, and other factors. TyG was consistently associated with the left atrial functional and coupling phenotypes LACI, LASr, and LASI, but not with LAVI or LVMI, suggesting that insulin-resistance burden may be more closely related to early functional impairment than to established chamber remodeling in this cohort. This interpretation is consistent with previous evidence linking altered left atrial mechanics to NT-proBNP and identifying early left atrial mechanical abnormalities in diabetes even when left atrial size is normal (22, 23). Possible mechanisms include chronic inflammation, endothelial dysfunction, lipotoxicity, myocardial metabolic stress, and microvascular injury; however, these mechanisms were not directly measured in the present study.

The longer diabetes duration in the non-overweight group warrants cautious interpretation. Although diabetes duration was included in the multivariable models, prolonged diabetes may be accompanied by disease-related weight loss, reduced muscle mass, malnutrition, or altered fat distribution, potentially leading to exposure misclassification when BMI is used alone. Conversely, the substantially greater waist circumference in the overweight/obesity group supports a higher central-adiposity burden, although waist circumference cannot distinguish visceral from subcutaneous adipose tissue. These findings indicate that the non-overweight group should not automatically be regarded as metabolically healthier. Because direct measures of muscle mass, muscle function, nutritional status, and regional fat distribution were unavailable, sarcopenia and visceral adiposity could not be formally evaluated. The possibility of a lean diabetes phenotype with sarcopenic or catabolic features therefore remains a hypothesis for future studies rather than a conclusion of this analysis.

### 4.4 Treatment patterns and robustness of the associations

Medication patterns should be interpreted in the context of treatment selection. GLP-1 receptor agonist use was more frequent in participants with BMI >=24 kg/m2, whereas insulin use was less frequent. This distribution is clinically plausible because GLP-1 receptor agonists are preferentially considered in adults with T2DM and overweight/obesity when weight reduction and cardiometabolic risk reduction are treatment goals (32). It therefore likely reflects prescribing according to baseline adiposity and metabolic risk, that is, confounding by indication, rather than a causal medication effect. The cross-sectional data cannot determine whether GLP-1 receptor agonists modified the echocardiographic phenotype or clinical outcomes. Nevertheless, after separate additional adjustment for GLP-1 receptor agonists, SGLT2 inhibitors, insulin, diuretics/mineralocorticoid receptor antagonists, and renin-angiotensin system inhibitors, the association between overweight/obesity and Layer 2 abnormality remained stable, with odds ratios ranging from 4.19 to 4.99. The association also persisted after excluding participants with eGFR <60 mL/min/1.73 m2. Medication-adjusted continuous-outcome models preserved the directions of association for BMI and TyG with LACI and for TyG with LASr and LASI. These findings support, but do not prove, the robustness of the observed associations.

### 4.5 Interpretation within the pre-heart failure and CKM frameworks

The findings can be interpreted within the updated pre-heart failure and cardiovascular-kidney-metabolic frameworks. Participants with NT-proBNP <125 pg/mL who had Layer 1 or Layer 2 echocardiographic abnormalities exhibited imaging features compatible with early, asymptomatic cardiac involvement and, in some cases, with the structural or functional phenotype of AHA Stage B heart failure. Although these findings do not establish a formal Stage B diagnosis because symptoms, invasive filling pressures, and all required staging variables were not systematically available, they indicate that the conventional NT-proBNP threshold alone may fail to identify some patients with pre-heart failure features. From a CKM perspective, T2DM, excess adiposity, insulin resistance, and kidney dysfunction represent interconnected Stage 2 metabolic and renal risk factors, whereas left atrial enlargement, impaired atrial strain, and adverse atrioventricular coupling may reflect subclinical cardiovascular involvement corresponding to the Stage 2-to-Stage 3 transition (25). The coexistence of an adverse echocardiographic phenotype with low NT-proBNP is therefore consistent with the possibility that metabolic disturbances may precede detectable natriuretic peptide elevation and contribute to early cardiac remodeling. These findings support the complementary use of echocardiographic phenotyping when conventional natriuretic peptide thresholds appear reassuring, while acknowledging that formal CKM staging was not performed.

Beyond natriuretic peptides and echocardiographic imaging, emerging molecular and inflammatory biomarkers may provide complementary information on early cardiometabolic injury. In individuals with prediabetes or newly diagnosed T2DM without established cardiovascular disease, several circulating long non-coding RNAs were associated with NT-proBNP, while ARL4C and NOS2P3 were also associated with epicardial adipose tissue thickness, suggesting a potential relationship between molecular biomarkers, adiposity-related changes, and early myocardial stress (33). In a separate high-risk population with familial hypercholesterolemia, the neutrophil-to-lymphocyte ratio was independently associated with subclinical atherosclerosis, supporting systemic inflammation as an additional dimension of subclinical cardiovascular risk assessment (34). These biomarkers were not measured in the present cohort, and their incremental value beyond NT-proBNP and echocardiographic phenotyping in patients with T2DM requires prospective evaluation.

### 4.6 Clinical value, strengths, and limitations

This study suggests that NT-proBNP <125 pg/mL alone should not be interpreted as excluding early cardiac involvement in patients with T2DM, particularly when excess or central adiposity, elevated TyG or METS-IR, or long diabetes duration is present. A complementary assessment incorporating structural measures and left atrial phenotyping may help identify patients with discordant biomarker and imaging profiles. The strengths of this study include the close timing of NT-proBNP measurement and echocardiography, the use of two prespecified echocardiographic layers, simultaneous evaluation of conventional and advanced phenotypes, and the use of Firth penalized logistic regression and HC3 robust standard errors. These findings should be viewed as hypothesis-generating for prospective risk-stratification studies rather than as evidence for a new diagnostic threshold.

This study has several limitations. First, the single-center stable inpatient sample may be subject to referral and selection bias, and the findings cannot be directly generalized to community screening settings or symptomatic outpatient HFpEF populations. Second, the retrospective cross-sectional design prevents assessment of temporal ordering, causality, progression to symptomatic heart failure, or formal AHA/CKM staging. Third, NT-proBNP was measured once, and invasive filling pressure and independent clinical outcomes were not used as reference standards. Fourth, medication exposure was assessed cross-sectionally; information on treatment duration, dose, adherence, and longitudinal treatment changes was unavailable, so residual confounding by treatment indication cannot be excluded despite the prespecified sensitivity analyses. Fifth, direct measures of skeletal muscle mass, muscle strength, nutritional status, and regional fat distribution were unavailable, preventing formal evaluation of sarcopenia or visceral adiposity. Sixth, exercise tolerance and heart-failure symptoms were not systematically collected, preventing symptom-stratified analysis. Finally, external validation was lacking, and the thresholds and association estimates require replication in larger, multicenter, prospective cohorts.

## 5 Conclusions

In hospitalized patients with type 2 diabetes mellitus, NT-proBNP <125 pg/mL did not fully exclude prespecified structural or diastolic echocardiographic abnormalities. Higher BMI and TyG were associated with adverse left atrial and atrioventricular coupling phenotypes, with these associations generally persisting after adjustment for major clinical factors and therapies. NT-proBNP should therefore be interpreted as one component of multimodal assessment rather than as a stand-alone rule-out tool for early cardiac involvement.

## Data Availability

The data analyzed in this study is subject to the following licenses/restrictions: The raw clinical datasets generated from this study are not publicly available due to patient−privacy and hospital−ethics restrictions. De−identified aggregated data may be available from the corresponding author upon reasonable request with formal ethics approval. Requests to access these datasets should be directed to zhaoyueyaocc@163.com.
