# Supplementary material for: NT-proBNP–echocardiographic discordance in type 2 diabetes: links with adiposity and left atrioventricular coupling
Source: Front Endocrinol (Lausanne). 2026 Sep 25;17:1971124. doi: 10.3389/fendo.2026.1971124 (PMC13649424; doi:10.3389/fendo.2026.1971124)
Supplement: Supplementary file 1 [file DataSheet1.pdf]

Supplementary Table S1. Intraobserver and interobserver reproducibility of echocardiographic measurements

Measurement	Intraobserver ICC (95% CI)	Intraobserver bias	Intraobserver 95% LoA	Interobserver ICC (95% CI)	Interobserver bias	Interobserver 95% LoA
Average E/e′	0.855 (0.724–0.954)	0.069	-1.972 to 2.111	0.873 (0.795–0.929)	-0.307	-2.165 to 1.550
2D LVM, g	0.933 (0.876–0.969)	-6.203	-25.966 to 13.559	0.881 (0.776–0.928)	-9.120	-31.959 to 13.719
2D LASr, %	0.978 (0.954–0.990)	-0.784	-6.138 to 4.569	0.940 (0.892–0.966)	0.148	-9.846 to 10.141
3D LVEDV, mL	0.972 (0.944–0.986)	-0.555	-10.405 to 9.295	0.947 (0.899–0.970)	-0.284	-15.102 to 14.535
3D LAVmax, mL	0.987 (0.961–0.995)	-1.733	-8.423 to 4.957	0.974 (0.922–0.988)	-0.467	-10.404 to 9.471
3D LAVmin, mL	0.987 (0.949–0.995)	-0.527	-6.040 to 4.986	0.983 (0.919–0.992)	-1.531	-7.391 to 4.330
LACI	0.989 (0.877–0.996)	-0.003	-0.063 to 0.057	0.970 (0.833–0.976)	-0.008	-0.116 to 0.100
LASI	0.912 (0.819–0.968)	0.009	-0.078 to 0.096	0.855 (0.724–0.929)	0.006	-0.123 to 0.134

Notes: Values are based on 30 randomly selected participants. ICCs and 95% CIs were estimated using a two-way absolute-agreement model. Bland-Altman differences were calculated as the second measurement minus the first measurement. ICC, intraclass correlation coefficient; CI, confidence interval; LoA, limits of agreement; 2D, two-dimensional; 3D, three-dimensional; LVM, left ventricular mass; LASr, left atrial reservoir strain; LVEDV, left ventricular end-diastolic volume; LAV, left atrial volume; LACI, left atrioventricular coupling index; LASI, left atrial stiffness index.

Supplementary Figure S1. Reproducibility of echocardiographic measurements

Bland-Altman plots for intraobserver (A) and interobserver (B) reproducibility. Each point represents one participant. Differences were calculated as the second measurement minus the first measurement. Blue lines indicate the mean bias, and orange lines indicate the 95% limits of agreement.

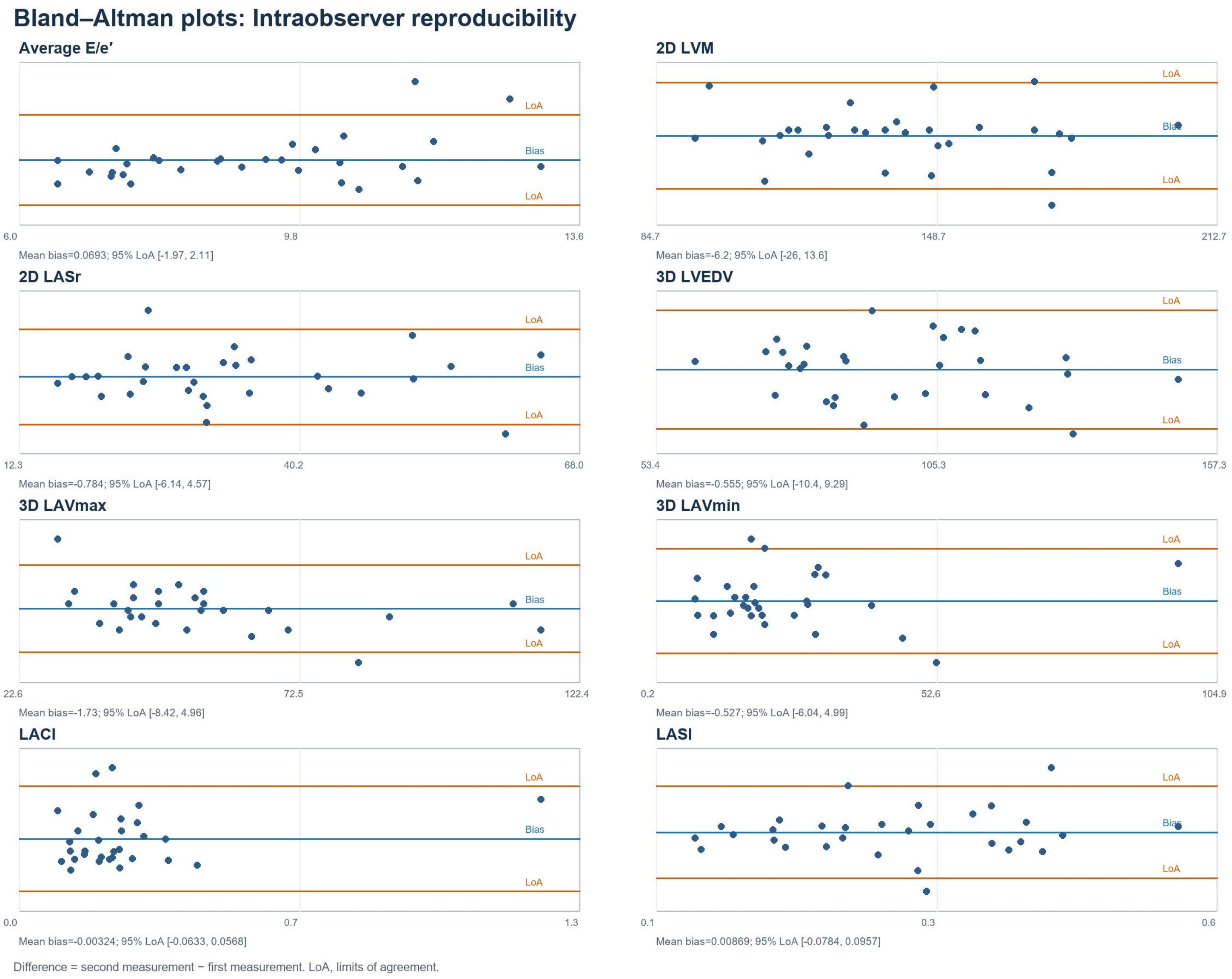

Supplementary Figure S1 continued: Interobserver reproducibility

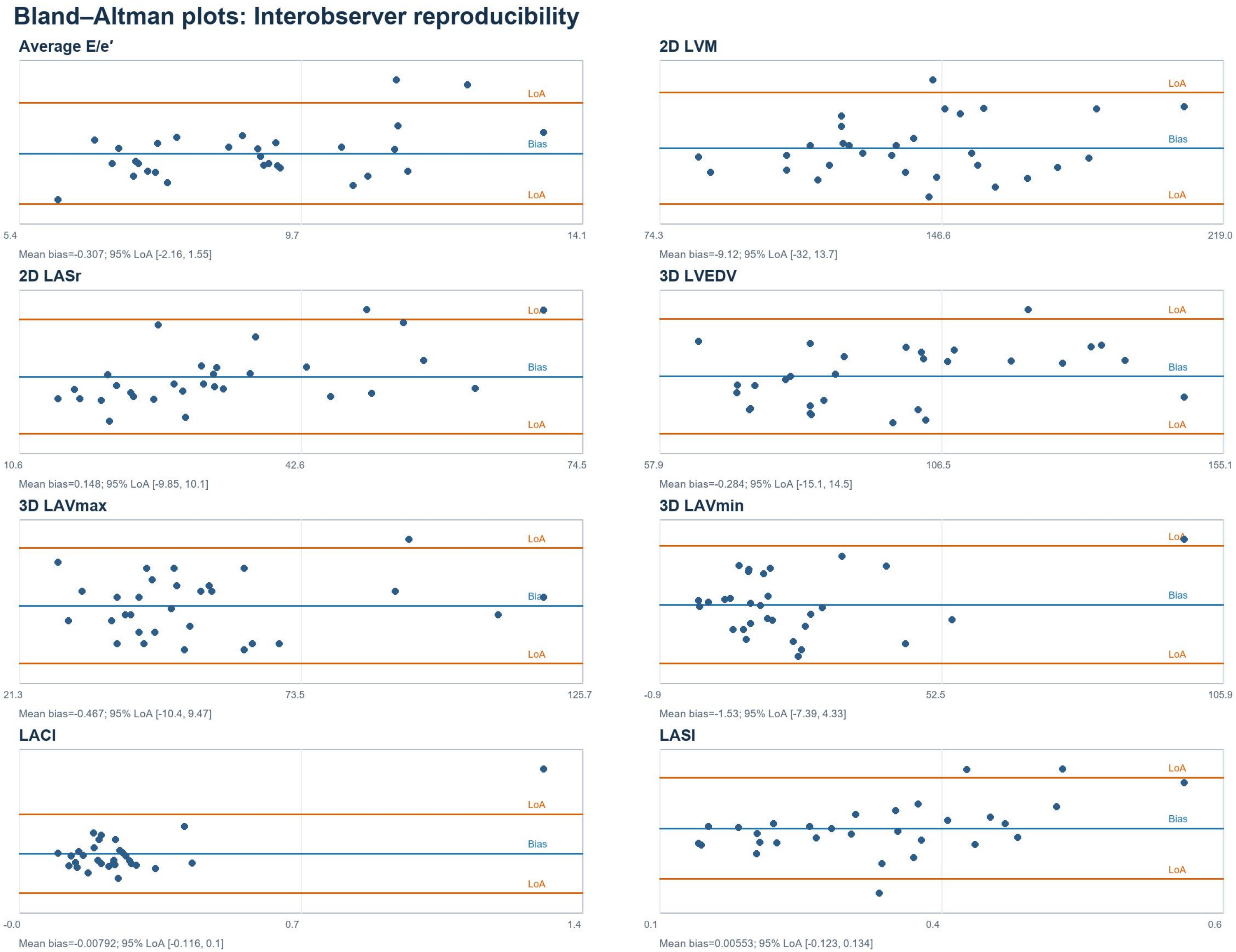

Difference = second measurement − first measurement. LoA, limits of agreement.

Supplementary Table S2. Component contributions and mutually exclusive patterns of the Layer 2 outcome

Component or pattern	Overall Layer 2 positive (n=63)	NT-proBNP <125 pg/mL (n=38)	NT-proBNP ≥125 pg/mL (n=25)
**A. Non-mutually-exclusive components**			
Layer 1 abnormality	33 (52.4)	17 (44.7)	16 (64.0)
Left atrial volume index >34 mL/m²	46 (73.0)	26 (68.4)	20 (80.0)
Elevated left ventricular mass index	22 (34.9)	14 (36.8)	8 (32.0)
Structural abnormality without Layer 1 abnormality	30 (47.6)	21 (55.3)	9 (36.0)
**B. Mutually exclusive patterns**			
Layer 1 only	3 (4.8)	1 (2.6)	2 (8.0)
Elevated LAVI only	14 (22.2)	9 (23.7)	5 (20.0)
Elevated LVMI only	13 (20.6)	10 (26.3)	3 (12.0)
Layer 1 + Elevated LAVI only	24 (38.1)	14 (36.8)	10 (40.0)
Layer 1 + Elevated LVMI only	1 (1.6)	1 (2.6)	0 (0.0)
Elevated LAVI + Elevated LVMI only	3 (4.8)	2 (5.3)	1 (4.0)
Layer 1 + Elevated LAVI + Elevated LVMI only	5 (7.9)	1 (2.6)	4 (16.0)

Notes: Values are n (%), with the number of Layer 2-positive participants in each column as the denominator. Percentages in panel A do not sum to 100% because components overlap. Structural abnormality without Layer 1 abnormality was defined as elevated LAVI and/or LVMI in the absence of Layer 1 abnormality. Elevated LVMI was defined as >95 g/m² in women and >115 g/m² in men. NT-proBNP, N-terminal pro-B-type natriuretic peptide; LAVI, left atrial volume index; LVMI, left ventricular mass index.

Supplementary Table S3. Glucose-lowering and cardiovascular medication use in the study cohort

A. Overall cohort according to NT-proBNP classification

Medication	Overall (n=191)	NT-proBNP <125 pg/mL (n=153)	NT-proBNP ≥125 pg/mL (n=38)	P value	Effect size
Sodium-glucose cotransporter 2 inhibitor	87 (45.5)	75 (49.0)	12 (31.6)	0.053	-0.36
Glucagon-like peptide-1 receptor agonist	45 (23.6)	41 (26.8)	4 (10.5)	0.034	-0.43
Insulin	100 (52.4)	79 (51.6)	21 (55.3)	0.688	0.07
Metformin	108 (56.5)	93 (60.8)	15 (39.5)	0.018	-0.44
Renin-angiotensin system inhibitor	45 (23.6)	34 (22.2)	11 (28.9)	0.382	0.15
Beta-blocker	28 (14.7)	19 (12.4)	9 (23.7)	0.079	0.3
Calcium channel blocker	46 (24.1)	33 (21.6)	13 (34.2)	0.103	0.28
Statin	125 (65.4)	100 (65.4)	25 (65.8)	0.960	0.01
Any antiplatelet agent	24 (12.6)	19 (12.4)	5 (13.2)	1.000	0.02
Dipeptidyl peptidase-4 inhibitor	31 (16.2)	29 (19.0)	2 (5.3)	0.041	-0.43
Alpha-glucosidase inhibitor	34 (17.8)	29 (19.0)	5 (13.2)	0.403	-0.16
Diuretic or mineralocorticoid receptor antagonist	8 (4.2)	2 (1.3)	6 (15.8)	<0.001	0.54
Other glucose-lowering medication	18 (9.4)	12 (7.8)	6 (15.8)	0.209	0.25

B. NT-proBNP <125 pg/mL subgroup according to body mass index

Medication	Body mass index <24 kg/m² (n=67)	Body mass index ≥24 kg/m² (n=86)	P value	Effect size
Sodium-glucose cotransporter 2 inhibitor	30 (44.8)	45 (52.3)	0.354	0.15
Glucagon-like peptide-1 receptor agonist	6 (9.0)	35 (40.7)	<0.001	0.79
Insulin	42 (62.7)	37 (43.0)	0.016	-0.4
Metformin	40 (59.7)	53 (61.6)	0.809	0.04
Renin-angiotensin system inhibitor	13 (19.4)	21 (24.4)	0.459	0.12
Beta-blocker	8 (11.9)	11 (12.8)	0.874	0.03
Calcium channel blocker	12 (17.9)	21 (24.4)	0.332	0.16
Statin	42 (62.7)	58 (67.4)	0.540	0.1
Any antiplatelet agent	9 (13.4)	10 (11.6)	0.737	-0.05
Dipeptidyl peptidase-4 inhibitor	14 (20.9)	15 (17.4)	0.589	-0.09
Alpha-glucosidase inhibitor	19 (28.4)	10 (11.6)	0.009	-0.43
Diuretic or mineralocorticoid receptor antagonist	2 (3.0)	0 (0.0)	0.190	-0.25
Other glucose-lowering medication	8 (11.9)	4 (4.7)	0.096	-0.27

Notes: Values are n (%). P values were obtained using the Pearson chi-square test or Fisher exact test, as appropriate, and were not adjusted for multiple comparisons. Effect sizes are standardized differences in proportions and are oriented as the second comparison group minus the first; positive values indicate a higher prevalence in the second group. Medication comparisons are descriptive and should not be interpreted as treatment effects. NT-proBNP, N-terminal pro-B-type natriuretic peptide.

Supplementary Table S4. Sensitivity analyses accounting for medication use, NT-proBNP concentration, and renal function

A. Firth penalized logistic regression sensitivity analyses

Outcome	Sensitivity model	n	Events	OR (95% CI)	P value
Layer 1 abnormality	Primary model	153	17	3.16 (1.03 to 9.74)	0.045
Layer 1 abnormality	+ log10 NT-proBNP	153	17	3.07 (1.00 to 9.40)	0.050
Layer 1 abnormality	+ SGLT2 inhibitor	153	17	3.20 (1.04 to 9.84)	0.042
Layer 1 abnormality	+ GLP-1 receptor agonist	153	17	3.01 (0.92 to 9.83)	0.068
Layer 1 abnormality	+ insulin	153	17	3.17 (1.03 to 9.73)	0.043
Layer 1 abnormality	+ diuretic or MRA	153	17	3.07 (1.00 to 9.45)	0.050
Layer 1 abnormality	+ RAS inhibitor	153	17	3.06 (0.99 to 9.46)	0.052
Layer 1 abnormality	eGFR ≥60 mL/min/1.73 m² subgroup	145	16	2.85 (0.91 to 8.96)	0.073
Layer 2 abnormality	Primary model	153	38	4.15 (1.66 to 10.39)	0.002
Layer 2 abnormality	+ log10 NT-proBNP	153	38	4.99 (1.90 to 13.07)	0.001
Layer 2 abnormality	+ SGLT2 inhibitor	153	38	4.19 (1.67 to 10.56)	0.002
Layer 2 abnormality	+ GLP-1 receptor agonist	153	38	4.85 (1.83 to 12.89)	0.002
Layer 2 abnormality	+ insulin	153	38	4.22 (1.68 to 10.58)	0.002
Layer 2 abnormality	+ diuretic or MRA	153	38	4.59 (1.78 to 11.82)	0.002
Layer 2 abnormality	+ RAS inhibitor	153	38	4.21 (1.66 to 10.66)	0.002
Layer 2 abnormality	eGFR ≥60 mL/min/1.73 m² subgroup	145	35	4.10 (1.54 to 10.88)	0.005

Notes: Firth penalized logistic regression estimated the association of BMI ≥24 kg/m² (reference, BMI <24 kg/m²) with each outcome among participants with NT-proBNP <125 pg/mL. The primary model included age, sex, diabetes duration, hypertension, eGFR, and HbA1c. Log10-transformed NT-proBNP and each of the five prespecified therapies were added separately. This parsimonious approach was used consistently across outcomes, particularly because Layer 1 included only 17 events. The renal-function sensitivity analysis excluded participants with eGFR <60 mL/min/1.73 m². OR, odds ratio; CI, confidence interval; BMI, body mass index; eGFR, estimated glomerular filtration rate; HbA1c, glycated hemoglobin; SGLT2, sodium-glucose cotransporter 2; GLP-1, glucagon-like peptide-1; MRA, mineralocorticoid receptor antagonist; RAS, renin-angiotensin system.

B. Linear regression sensitivity analyses

Outcome	Sensitivity model	Exposure	n	Standardized β (95% CI)	P value	R^2
LACI	+ five therapies	BMI	153	0.22 (0.09 to 0.35)	0.001	0.180
LACI	+ five therapies	TyG	153	0.18 (0.05 to 0.31)	0.006	0.180
LACI	+ log10 NT-proBNP	BMI	153	0.22 (0.09 to 0.35)	<0.001	0.145
LACI	+ log10 NT-proBNP	TyG	153	0.16 (0.05 to 0.27)	0.004	0.145
LACI	eGFR ≥60 mL/min/1.73 m² subgroup	BMI	145	0.35 (0.13 to 0.57)	0.002	0.239
LACI	eGFR ≥60 mL/min/1.73 m² subgroup	TyG	145	0.25 (0.09 to 0.42)	0.003	0.239
2D LASr	+ five therapies	BMI	153	-0.15 (-0.33 to 0.04)	0.122	0.211
2D LASr	+ five therapies	TyG	153	-0.24 (-0.45 to -0.03)	0.022	0.211
2D LASr	+ log10 NT-proBNP	BMI	153	-0.10 (-0.27 to 0.07)	0.244	0.154
2D LASr	+ log10 NT-proBNP	TyG	153	-0.25 (-0.46 to -0.05)	0.015	0.154
2D LASr	eGFR ≥60 mL/min/1.73 m² subgroup	BMI	145	-0.10 (-0.28 to 0.09)	0.308	0.155
2D LASr	eGFR ≥60 mL/min/1.73 m² subgroup	TyG	145	-0.21 (-0.41 to -0.01)	0.037	0.155
LASI	+ five therapies	BMI	153	0.22 (0.03 to 0.40)	0.022	0.183
LASI	+ five therapies	TyG	153	0.27 (0.08 to 0.47)	0.007	0.183
LASI	+ log10 NT-proBNP	BMI	153	0.16 (-0.01 to 0.32)	0.059	0.165
LASI	+ log10 NT-proBNP	TyG	153	0.29 (0.10 to 0.48)	0.003	0.165
LASI	eGFR ≥60 mL/min/1.73 m² subgroup	BMI	145	0.15 (-0.02 to 0.33)	0.077	0.159
LASI	eGFR ≥60 mL/min/1.73 m² subgroup	TyG	145	0.26 (0.06 to 0.45)	0.011	0.159

Notes: All models included BMI, TyG, age, sex, diabetes duration, hypertension, eGFR, and HbA1c. The medication-adjusted model additionally included SGLT2 inhibitor, GLP-1 receptor agonist, insulin, diuretic or MRA, and RAS inhibitor use. Continuous NT-proBNP was log10 transformed. Continuous exposures and outcomes were standardized, and HC3 heteroskedasticity-robust standard errors were used. BMI, body mass index; TyG, triglyceride-glucose index; eGFR, estimated glomerular filtration rate; HbA1c, glycated hemoglobin; LACI, left atrioventricular coupling index; LASr, left atrial reservoir strain; LASI, left atrial stiffness index; CI, confidence interval.
